# Chemical Replacement of Noggin with Dorsomorphin Homolog 1 for Cost-Effective Direct Neuronal Conversion

**DOI:** 10.1089/cell.2021.0200

**Published:** 2022-10-07

**Authors:** Lena Böhnke, Lucia Zhou-Yang, Silvia Pelucchi, Flora Kogler, Daniela Frantal, Florian Schön, Stina Lagerström, Oliver Borgogno, Jennifer Baltazar, Joseph R. Herdy, Sarah Kittel-Schneider, Michaela Defrancesco, Jerome Mertens

**Affiliations:** ^1^ Neural Aging Laboratory, Institute of Molecular Biology, CMBI, University of Innsbruck, Innsbruck, Tyrol, Austria.; ^2^ Laboratory of Genetics, The Salk Institute for Biological Studies, La Jolla, California, USA.; ^3^ Center of Mental Health, Department of Psychiatry, Psychosomatic Medicine and Psychotherapy, University Hospital Würzburg, Würzburg, Bavaria, Germany.; ^4^ Division of Psychiatry I, Department of Psychiatry and Psychotherapy, Medical University of Innsbruck, Innsbruck, Tyrol, Austria.

**Keywords:** DMH1, Noggin, direct conversion, induced neurons, culture media

## Abstract

The direct conversion of adult human skin fibroblasts (FBs) into induced neurons (iNs) represents a useful technology to generate donor-specific adult-like human neurons. Disease modeling studies rely on the consistently efficient conversion of relatively large cohorts of FBs. Despite the identification of several small molecular enhancers, high-yield protocols still demand addition of recombinant Noggin. To identify a replacement to circumvent the technical and economic challenges associated with Noggin, we assessed dynamic gene expression trajectories of transforming growth factor-β signaling during FB-to-iN conversion. We identified ALK2 (ACVR1) of the bone morphogenic protein branch to possess the highest initial transcript abundance in FBs and the steepest decline during successful neuronal conversion. We thus assessed the efficacy of dorsomorphin homolog 1 (DMH1), a highly selective ALK2-inhibitor, for its potential to replace Noggin. Conversion media containing DMH1 (+DMH1) indeed enhanced conversion efficiencies over basic SMAD inhibition (tSMADi), yielding similar βIII-tubulin (TUBB3) purities as conversion media containing Noggin (+Noggin). Furthermore, +DMH1 induced high yields of iNs with clear neuronal morphologies that are positive for the mature neuronal marker NeuN. Validation of +DMH1 for iN conversion of FBs from 15 adult human donors further demonstrates that Noggin-free conversion consistently yields iN cultures that display high βIII-tubulin numbers with synaptic structures and basic spontaneous neuronal activity at a third of the cost.

## Introduction

Aging is one of the most significant risk factors for many neurodegenerative diseases, and establishing predictive models for disease modeling and drug discovery that reflect aspects of old adult human brain cells is a challenge. The majority of cell-based models rely on differentiation from human induced pluripotent stem cells (iPSCs), but during reprogramming of human somatic cells into iPSCs, the cells undergo a process of global cellular rejuvenation to early embryonic stages and lose signatures of aging (Lapasset et al., 2011; Miller et al., 2013; Sardo et al., 2017; Takahashi et al., 2007).

As a consequence, they closely resemble embryonic, but lack signatures of adultness and age, which is a limitation when modeling age-dependent diseases (Böhnke et al., 2018; Miller et al., 2013; Vera and Studer, 2015). As opposed to neurons derived from iPSCs, directly converted induced neurons (iNs) from human donor fibroblasts (FBs) retain many transcriptomic, molecular, metabolic, and epigenetic aging signatures (Böhnke et al., 2018; Chambers et al., 2009; Huh et al., 2016; Kim et al., 2018; Mertens et al., 2015; Pang et al., 2011), and they thus represent an attractive model system to study age-related neurological diseases in a patient- and cohort-specific manner (Drouin-Ouellet et al., 2021; Ma et al., 2020; Mertens et al., 2021; Victor et al., 2014).

While several protocols for direct conversion of FBs to iNs exist, most studies utilize the overexpression of pioneer transcription factors such as Achaete-scute homolog 1 (Ascl1) and Neurogenin 2 (Ngn2), which together have been shown to yield the highest and most reliable conversion efficiencies in combination with cocktails of small molecular pathway modulators (Ladewig et al., 2012; Liu et al., 2013). Most small molecules result in stimulation of proneuronal pathways such as cyclic AMP signaling, and downregulation of antineuronal pathways such as glycogen synthase kinase 3 beta (GSK3β), hypoxia-inducible factor 1-alpha (HIF1-α), integrin, or signal transducer and activator of transcription 3 signaling (STAT3) (Herdy et al., 2019; Ladewig et al., 2012; Liu et al., 2013).

One of these antineuronal pathways is transforming growth factor-β (TGFβ) superfamily signaling, which uses the tyrosine kinase receptors of the anaplastic lymphoma kinase (ALK) family to phosphorylate SMAD proteins to signal to the nucleus (Miyazono, 2000). TGFβ signaling can be divided into a TGFβ1 arm (ALK4/5/7 and SMAD2/3) and a bone morphogenic protein (BMP) arm (ALK2/3/6 and SMAD1/5/8). The proneuronal activity of the inhibition of both arms was first demonstrated in a landmark discovery by Chambers et al. (2009), describing that inhibition by the small molecule SB-431542 (ALK4/5/7 inhibitor) and the BMP antagonist Noggin induces highly efficient neuralization of human iPSCs, a method known as dual-SMAD inhibition (Chambers et al., 2009), and that strategy has been quickly adopted also for direct iN conversion (Ladewig et al., 2012; Liu et al., 2013; Vadodaria et al., 2016).

While several additional chemical ALK inhibitors, including A83-1 (ALK4/5/7), dorsomorphin (ALK2/3/6), and LDN-193189 (ALK2/3), have been used successfully, none of those was able to replace Noggin in terms of achieving efficient high-quality iN conversion (Herdy et al., 2019; Ladewig et al., 2012). However, due to their higher purity, lower cost, and less variable activity, small molecules are favored over recombinant proteins for larger cell reprogramming studies.

## Results and Discussion

### High FB abundance and pronounced repression of ALK2-mediated BMP signaling during successful iN conversion

Because TGFβ superfamily signaling pathways are instrumental in orchestrating the neuronal conversion process in various *in vivo* and *in vitro* systems (Falk et al., 2008; Li et al., 2011; Yin et al., 2019), we investigated the dynamic transcriptional trajectories of TGFβ signaling component genes during reprogramming in tSMADi plus Noggin condition (+Noggin). For this, we assessed gene expression trajectories of FB-to-iN conversion based on time series whole-transcriptome RNA-Seq data from FBs and converting cultures from three adult donors for 5, 10, 15, and 20 days (Herdy et al., 2019).

**FIG. 1.**
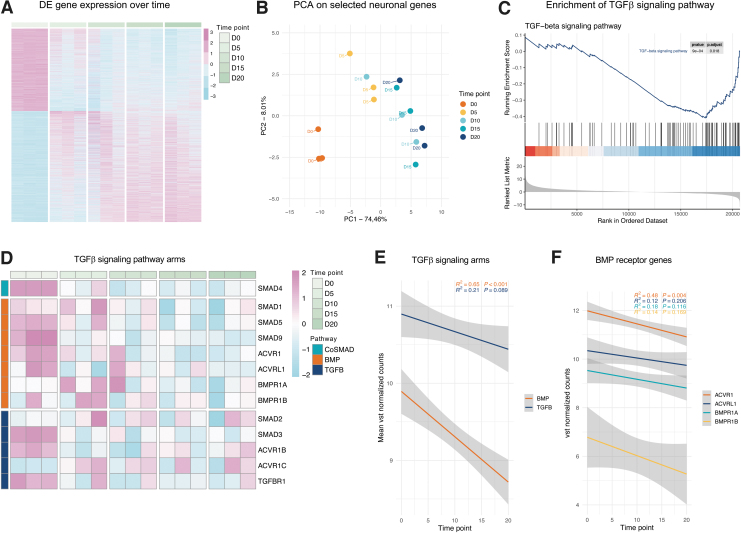
**(A)** Time series heatmap based on 4863 highly significant DEG (padj <0.01, 2075 downregulated and 2788 upregulated) on days 0, 5, 10, 15, and 20 of neuronal conversion. **(B)** PCA of 26 selected neuronal genes. Each *circle* is one subject at a certain time point (distinguished by *color*). **(C)** Enrichment plot of *TGF-beta signaling pathway* in GSEA differentially expressed between iNs and FBs. *Red*: positively correlated with iNs; *blue*: positively correlated with FBs. **(D)** Time series heatmap of 13 TGFβ signaling pathway receptor genes on days 0, 5, 10, 15, and 20 of neuronal conversion. *Orange*: BMP signaling arm; *dark blue*: TGFβ1 signaling arm; *turquoise*: Co-SMAD. **(E)** Regression analysis of mean expression values in BMP (*orange*) and TGFβ1 (*dark blue*) signaling arms on days 0, 5, 10, 15, and 20 of neuronal conversion. **(F)** Regression analysis of BMP signaling arm-specific receptor genes ACVR1 (*orange*), ACVRL1 (*dark blue*), BMPR1A (*turquoise*), and BMPR1B (*yellow*) on days 0, 5, 10, 15, and 20 of neuronal conversion. BMP, bone morphogenic protein; DEGs, differentially expressed genes; FBs, fibroblasts; GSEA, Gene Set Enrichment Analysis; iN, induced neuron; PCA, principal component analysis; TGFβ, transforming growth factor-β.

Comparison of FB and day 20 iN cultures identified 4863 highly significant differentially expressed genes (DEGs; padj <0.01). Of these 4863 DEGs, 2075 became downregulated and 2788 became upregulated during conversion, and the majority of which revealed a consistent and gradual repression or activation over time (Fig. 1A). Principal component analysis (PCA) of selected neuronal genes (Supplementary Table S1) and genes from Reactome pathway Neuronal System (R-HSA-112316) revealed that PC1 and PC2 together captured over 82% and 66% of the transcriptional variance, respectively, and illustrate a conversion trajectory along a combination of both components (Fig. 1B; Supplementary Fig. S1A–D).

The initial conversion trajectory (0–10 days) follows the loading vectors of basic neuronal genes such as neurofilament (*NEFL*) and βIII-tubulin (*TUBB3*), while later neuronal consolidation (10–20 days) follows loading vectors of AMPA and GABA receptor (*GRIA2* and *GABBR2*) and NeuN (*RBFOX3*) genes (Supplementary Fig. S1A). PC loadings of the Reactome pathway indicate changes toward neuronal identity for the majority of top-ranked genes during conversion (Supplementary Fig. S1D).

Gene Set Enrichment Analysis (GSEA) revealed a strong and significant enrichment of neuron-associated Kyoto Encyclopedia of Genes and Genomes (KEGG) pathways in iNs, including neuroactive ligand-receptor interaction (hsa04080), oxidative phosphorylation (hsa00190), and synaptic vesicle cycle (hsa04721), whereas pathways like cell cycle (hsa04110), adherens junction (hsa04520), and extracellular matrix-receptor (ECM) interaction (hsa04512) were enriched in FBs (Supplementary Fig. S1E, F; Supplementary Table S2).

Interestingly, genes of the TGFβ signaling pathway (hsa04350) were almost exclusively highly enriched in FBs (Fig. 1C). Because TGFβ signaling can be divided into two main arms, the TGFβ1 arm and the BMP arm, we extracted the respective receptor genes, ALKs and SMADs, which are specific for either of the two arms from the time series data in a heatmap and performed linear regression analyses (Fig. 1D–E). First, both signaling arms showed a significant repression over the time course of conversion, validating that pathway repression of both arms is indeed important for iN conversion (Fig. 1D–E). Variability between donors was assessed with mean standard deviation per time point, and indicated major changes during the first 10 days of conversion (Supplementary Fig. S1H).

In addition, the average gene expression of BMP arm showed a steeper and more significant repression than genes specific to the TGFβ1 arm (Fig. 1E), and among the BMP arm-specific components, ALK2 (*ACVR1*) showed the highest initial messenger RNA (mRNA) abundance in the FB stage, and the most significant drop in expression during conversion (Fig. 1F; Supplementary Fig. S1G). These data indicate that BMP signaling through ALK2 receptor kinase signaling might be a promising signaling component where targeted inhibition might be beneficial for direct iN conversion from adult human FBs.

### The ALK2-specific inhibitor dorsomorphin homolog 1 facilitates efficient iN conversion similar to Noggin on a tSMADi background

**FIG. 2.**
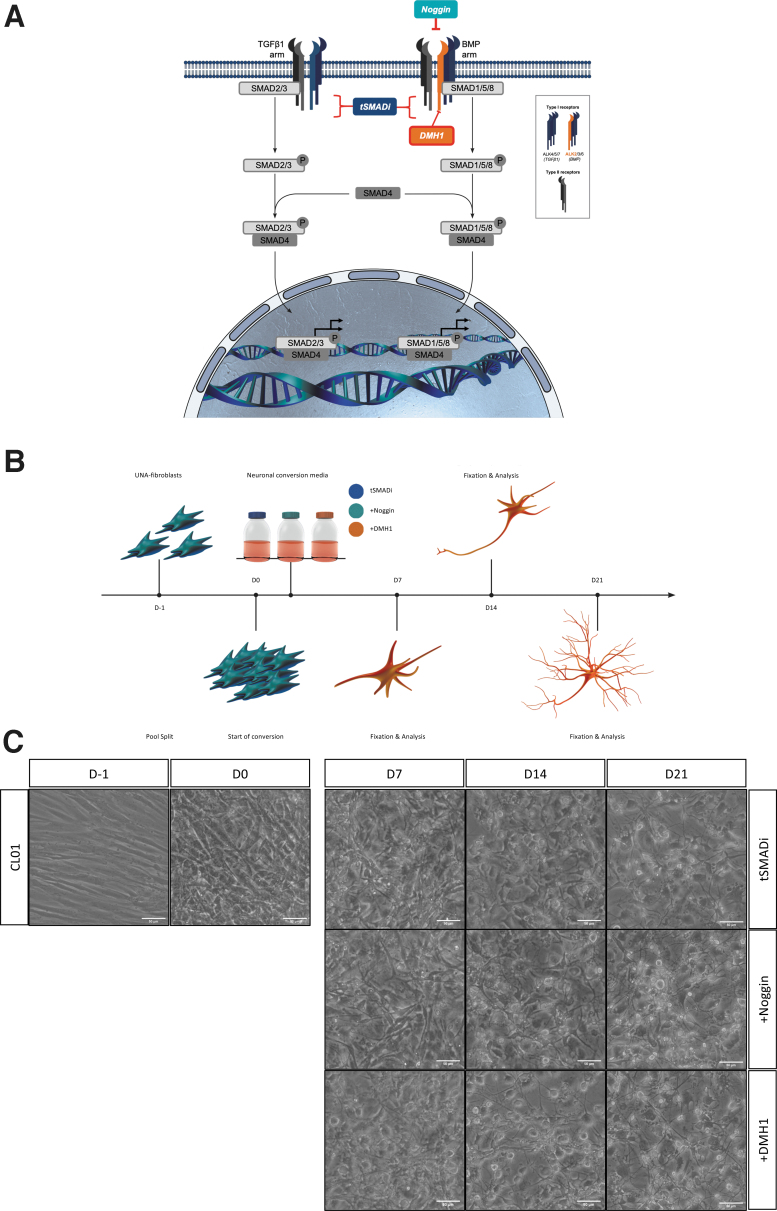
**(A)** TGFβ signaling pathway with TGFβ1 and BMP signaling arm-specific receptors and inhibitors tSMADi (*dark blue*), Noggin (*turquoise*), and DMH1 (*orange*). **(B)** Generation of fibroblast-derived iNs in three different neuronal conversion media (tSMADi, +Noggin, and +DMH1) over 21 days. **(C)** Brightfield images of CL01 before (D-1) and after (D0) pool split, and during (D7, D14, and D21) conversion in three different neuronal conversion media (tSMADi, +Noggin, and +DMH1). Scale bars: 50 μm. DMH1, dorsomorphin homolog 1.

In tSMADi, the compounds SB-431542 and A83-1 inhibit TGFβ arm-specific ALK4, ALK5, and ALK7 receptors, and thus are not blocking signaling through the BMP arm of TGFβ (Fig. 2A). Furthermore, the tSMADi compound LDN-193189, which is similar to but more potent than dorsomorphin, unspecifically targets ALK2, ALK3, and ALK6 receptors of the BMP arm (Fig. 2A). Because our time series transcriptome analysis suggests ALK2 inhibition to be the most promising target to enhance iN conversion, and tSMADi contains only LDN-193189 as a nonspecific ALK2 inhibitor in the absence of Noggin, we became interested in the specific ALK2 inhibitor dorsomorphin homolog 1 (DMH1) and its reported IC50s in the range of 10–100 nM (Hao et al., 2010; Mohedas et al., 2013; Neely et al., 2012).

To test the ALK2-specific inhibitor DMH1 for iN conversion, human adult FBs from two donors—CL01 (68 years, female) and CL02 (77 years, female)—were assessed. The cells were pooled into high densities in serum-containing dox-free media, and the media were changed to dox-containing iN conversion media containing tSMADi only (further referred to as tSMADi), tSMADi plus 100 ng/mL recombinant human Noggin (+Noggin), or tSMADi plus 5 μM DMH1 (+DMH1). 5 μM DMH1 in +DMH1 media was previously confirmed as the optimal concentration with a titration curve (Supplementary Fig. S2A).

**FIG. 3.**
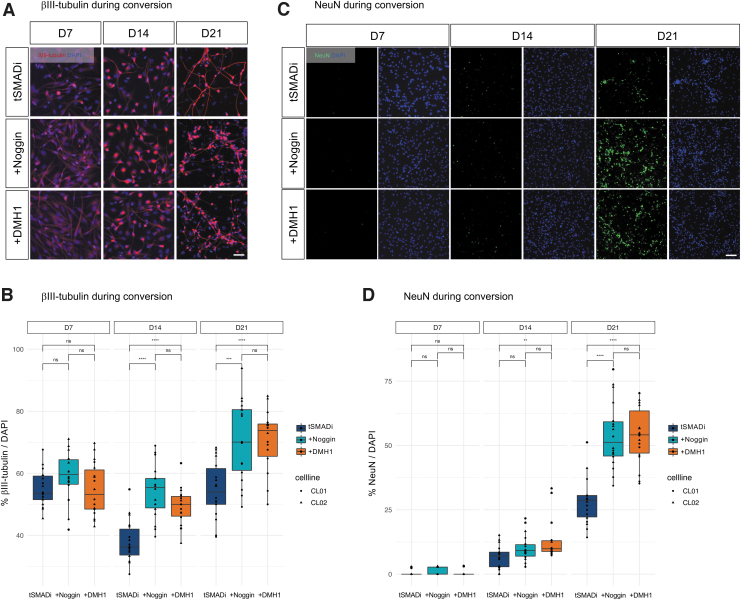
**(A)** Immunofluorescent images of CL01 on days 7, 14, and 21 of conversion. βIII-tubulin (*red*); DAPI (*blue*). **(B)** Quantification of immunofluorescent images of CL01 and CL02 for βIII-tubulin/DAPI on 7, 14, and 21 days of conversion. **(C)** Immunofluorescent images of CL01 on 7, 14, and 21 days of conversion. NeuN (*green*); DAPI (*blue*). **(D)** Quantification of immunofluorescent images of CL01 and CL02 for NeuN/DAPI on 7, 14, and 21 days of conversion. Scale bars: 50 μm; significance: unpaired *t*-test; ns: *p* > 0.05; **: *p* ≤ 0.01; ***: *p* ≤ 0.001, ****: *p* ≤ 0.0001.

We observed that iNs formed in all three conditions, and that basal levels of toxicity did not appear to be elevated in any of the three conditions (Fig. 2C; Supplementary Fig. S2B). To quantitatively compare +DMH1 to +Noggin and tSMADi, the cells were fixed and analyzed at 7, 14, and 21 days of conversion and stained for the neuronal marker βIII-tubulin. All three conditions yielded iNs that displayed clear neuronal morphologies (Fig. 3A; Supplementary Fig. S3A), and quantification revealed that at 14 and 21 days of conversion, +DMH1 and +Noggin significantly produced βIII-tubulin-positive cells, and +DMH1 showed less variability than +Noggin (Fig. 3B).

We next stained both lines with the mature neuron-specific marker NeuN (Fig. 3C; Supplementary Fig. S3B), and found that both +DMH1 and +Noggin yielded approximately twofold more NeuN-positive iNs at 21 days than tSMADi, and only +DMH1 produced significantly more NeuN cells already at 14 days (Fig. 3D). These data suggest that +DMH1 and +Noggin are particularly important to generate fully converted cells from intermediate conversion stages. We thus asked whether +DMH1 and +Noggin can equally boost the transition of cells with intermediate transition morphologies toward fully converted iNs with mature neuronal morphologies.

**FIG. 4.**
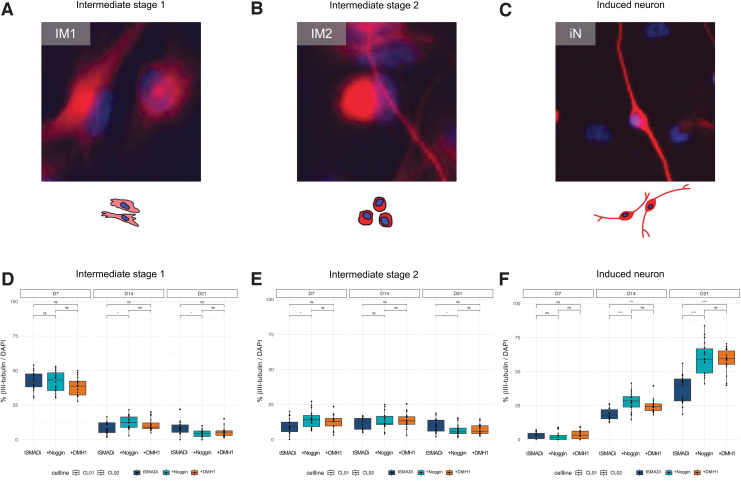
**(A)** Cell type of IM1. Weak βIII-tubulin staining, no neuronal morphology. **(B)** Cell type of IM2. Strong βIII-tubulin staining, no neuronal morphology. **(C)** iN. Strong βIII-tubulin staining, neuronal morphology. **(D–F)** Quantification of immunofluorescent images of IM1 **(D)**, IM2 **(E),** and iNs **(F)** in CL01 and CL02 for βIII-tubulin/DAPI during conversion. Significance: *t*-test; *: *p* ≤ 0.05; ***: *p* ≤ 0.01; ****: *p* ≤ 0.001. Staining: βIII-tubulin (*red*), DAPI (*blue*). IM1, intermediate stage 1; IM2, intermediate stage 2.

We classified βIII-tubulin-positive cells into three types (Fig. 4A–C): intermediate stage 1 (IM1, weak staining and no neuronal morphology), intermediate stage 2 (IM2, strong staining and no neuronal morphology), and neuron stage (iN, strong staining and neuronal morphology). Quantification confirmed that neither +DMH1 nor +Noggin had a strong and consistent effect on the formation of IM1 or IM2 cells (Fig. 4D, E), but specifically and significantly increased the number of iNs at 14 and 21 days (Fig. 4F). These data demonstrate that the small molecule DMH1 enhances the direct conversion of adult human FBs into mature iNs equally to recombinant Noggin, and that +DMH1 conversion is slightly less variable and specifically supports later stages of neuronal conversion.

### Consistent and economic production of high-quality iNs from a cohort of adult human FB using +DMH1.

Comparative iN-based disease modeling studies are based on relatively large cohorts of patient FBs, and demand several rounds of efficient and reliable conversion efficiencies into iNs with mature neuronal properties. Despite the use of LDN-193189 and other ALK inhibitors, many such studies that have assessed large FB cohorts critically depend on recombinant Noggin, which, however, is variable and expensive (Drouin-Ouellet et al., 2021; Mertens et al., 2021; Pircs et al., 2021).

**FIG. 5.**
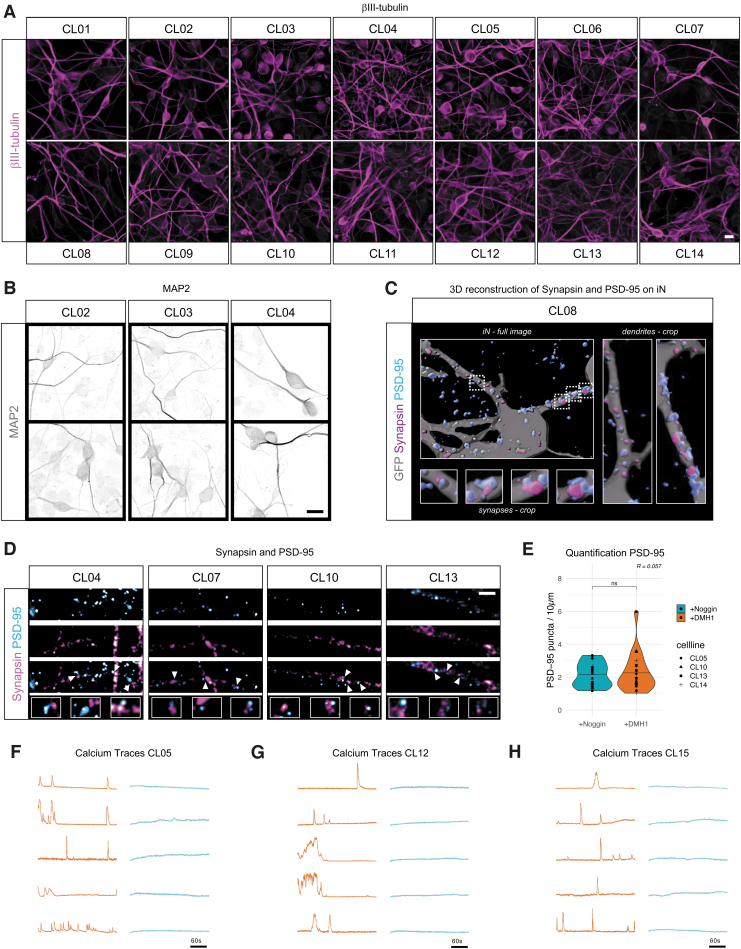
**(A)** Immunofluorescent images of CL01–CL14 on D21 of conversion. βIII-tubulin (*magenta*). Scale bar: 20 μm. **(B)** Immunofluorescent images of CL02, CL03, and CL04 on D21 of conversion. MAP2 (*grey*), scale bar = 15 μm. **(C)** Immunofluorescent images of CL01 and CL02 on D21 of conversion. Synapsin (*magenta*) and PSD-95 (*cyan*), scale bar = 2 μm. *Arrows* indicate close proximity of synapsin and PSD-95. **(D)** 3D reconstruction of LVXTP-GFP-transduced iN (*grey*), stained with synapsin (*magenta*) and PSD-95 (*cyan*). Shown are a complete image of one iN, two crops of dendrites, and four crops of synapses in higher magnification. **(E)** Quantification of number of PSD-95 puncta per 10 μm in 4 cell lines. *p* = 0.73. **(F–H)** Normalized calcium imaging traces in CL05 **(E)**, CL12 **(G),** and CL15 **(F)**. Active iN (*orange*, *n* = 5); inactive iN (*blue*, *n* = 5); scale bars: 60 seconds.

To test if +DMH1 can indeed reliably yield iNs with high efficiency, dendritic morphologies, mature synaptic-like structures, and neuronal activity properties, we assessed FBs from 15 adult human donors (Supplementary Table S4). The FBs were converted into iNs using +DMH1, fixed and stained with βIII-tubulin at 21 days, and all 15 lines yielded healthy cultures of mature iNs with many of the cells being βIII-tubulin-positive (Fig. 5A). Staining with the mature dendritic marker MAP2 revealed complex dendritic networks, and the close proximity of synapsin puncta to the postsynaptic density (PSD) marker PSD-95, seen in the three-dimensional (3D) reconstruction and two-dimensional images, suggests the presence of synapses throughout these dendritic networks (Fig. 5B–D).

Quantification of the number of PSD-95 puncta in the dendritic networks of +Noggin and +DMH1-iNs showed similar densities (Fig. 5E). To assess spontaneous functional activity by live-cell somatic calcium imaging, we next lentivirally transduced the +DMH1-iNs with GCaMP6m and imaged the cells at 35 days of conversion. Importantly, and similar to previous studies based on iNs generated with +Noggin (Mertens et al., 2015, 2021), we observed frequent and spontaneous neuronal activity in all assessed iN cultures (Fig. 5F–H). +DMH1 thus allows for the consistent generation of mature, healthy, and functional iNs from a relatively large FB donor cohort, and facilitates studies that demand the routine production of high-quality iNs.

**FIG. 6.**
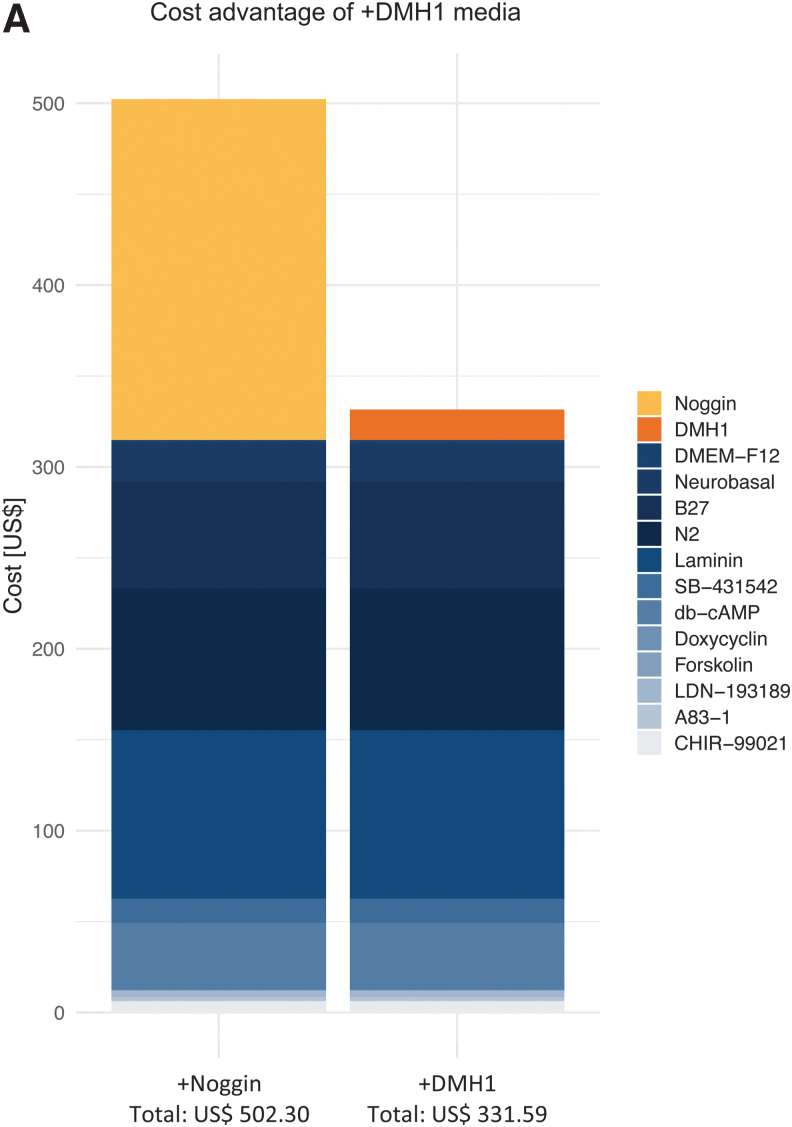
**(A)** Cost advantage of +DMH1 neuronal conversion medium over +Noggin.

In our conventional conversion media containing tSMADi and recombinant Noggin (+Noggin), Noggin is the by far the biggest cost factor accounting for 37% of the cost, followed by Laminin (18%), N2 supplement (16%), and B27 supplement (12%) (Supplementary Fig. S4). In this study, we could demonstrate that the small molecular ALK2 inhibitor DMH1 can replace recombinant Noggin at 10% of the cost, lowering conversion media costs by over 30%, and without sacrificing conversion efficiencies or iN quality (Fig. 6).

Our finding will be helpful to facilitate future cohort-based studies using iN technology, which is particularly useful for better understanding aspects of human neuronal cell aging (Huh et al., 2016; Mertens et al., 2015) and human age-related diseases such as ALS (Jovıˇcić et al., 2015), Huntington's disease (Pircs et al., 2021; Victor et al., 2018), Parkinson's disease (Drouin-Ouellet et al., 2021), or Alzheimer's disease (Ma et al., 2020; Mertens et al., 2021).

## Materials and Methods

### Bioinformatics analysis

RNA sequencing data from Herdy et al. (2019): libraries were prepared with TruSeq stranded mRNA sample prep kit (Illumina) and sequenced single-end 50 bp on Illumina HiSeq 2500 platform. Read trimming and mapping were performed using TrimGalore and STAR, respectively. Raw counts were generated using HTseq variance stabilizing transformation normalization (vst) and differential expression analysis was performed in DEseq2 with default Wald test for hypothesis testing. Cutoff for highly significant DEGs for this study was log2-fold change with a corresponding padj <0.01 (Herdy et al., 2019). Bioinformatics and statistics analyses of RNA-Seq data from Herdy et al. (2019) were performed with R version 4.1.1 and applied methods are indicated in figure legends. GSEA to compare gene expression changes (log2FC) between FBs and D20 iN in KEGG pathways was performed with R package *clusterProfiler*.

### Subjects

Human FB cell lines: CL01 (68 years, female), CL02 (77 years old, female), CL03 (55 years, female), CL04 (81 years, female), CL05 (79 years, female), CL06 (55 years, male), CL07 (79 years, female), CL08 (67 years, female), CL09 (74 years, male), CL10 (64 years, female), CL11 (55 years, female), CL12 (66 years, male), CL13 (66 years, male), CL14 (59 years, male), and CL15 (56 years, male). FB cultures were derived from small skin punch biopsies using standard procedures, and as previously described (Zhou-Yang et al., 2021). All participants provided written informed consent, and all procedures were approved by local human subject committees (EK No. 105312018).

### Cell culture

FBs were cultured in Dulbecco's Modified Eagle's Medium (DMEM) with 15% fetal bovine serum (FBS) and 1% NEAA (Thermo Fisher) and transduced with lentivirus pLVXUbC-rtTA-Ngn2:2A:Ascl1 (UNA, Addgene: No. 127289). UNA-FBs were selected with 1 μg/mL puromycin (Sigma) as previously described (Zhou-Yang et al., 2021). Conversion of UNA-FBs was initiated by a 3:1 pool split followed by a media change 24 hours later to one of the three neuronal conversion media tSMADi, +Noggin, or +DMH1.

All three media are based on DMEM:F12 and neurobasal (1:1, both Thermo Fisher), supplemented with N2 (1 × , ThermoFisher), B27 (1 × , ThermoFisher), doxycycline (2 μg/mL, Sigma-Aldrich), laminin (1 μg/mL, Sigma-Aldrich), dibutyryl-cyclic-AMP (100 μg/mL, Santa Cruz), LDN-193189 (0.5 μM, Sanova Pharma), A83-1 (0.5 μM, Santa Cruz), CHIR99021 (3 μM, LC Laboratories), forskolin (5 μM, LC Laboratories), and SB-431542 (10 μM, MedChem). +Noggin is additionally supplemented with human recombinant Noggin (100 ng/mL, R&D) and +DMH1 with DMH1 (5 μM, Sanova Pharma). Media were changed every 48 hours. After 7, 14, and 21 days of neuronal conversion, cells were subject to fixation and staining.

### Immunocytochemistry

Cells were fixed with 4% paraformaldehyde and stained in phosphate buffered saline (PBS) containing 0.05% Triton-X100 and 5% FBS. Primary antibodies (NeuN, 1:100, Merck Millipore; βIII-tubulin, 1:1000, BioLegend; MAP2, 1:750, abcam; PSD-95, 1:500, Antibodies Inc.; and synapsin I, 1:500, Calbiochem) were applied overnight at 4°C. Secondary antibodies [Donkey-Anti-Mouse IgG Alexa Fluor 488, Donkey-Anti-Rabbit IgG Alexa Fluor 647 (both Invitrogen); Donkey-Anti-Chicken IgG Cy3 (EMD Millipore); and CyTM3 AffiniPure Donkey-Anti-Rabbit IgG (H+L) (Jackson ImmunoResearch), all 1:250] were incubated for 2 hours at room temperature.

After DNA staining with DAPI (4′,6-diamidino-2-phenylindole 1:50,000) (Sigma-Aldrich) and PBS washing, slides were mounted in PVA-DABCO (polyvinyl alcohol mounting medium with DABCO^®^) (Merck). Stained cells were assessed on Leica DMi8 microscope with 10 × and 20 × objectives (NeuN, βIII-tubulin) and on Zeiss Cell Observer Spinning Disc Confocal Microscope (MAP2, PSD-95, and synapsin I) and analyzed with image processing package Fiji. βIII- tubulin and NeuN counts were normalized over DAPI (detailed information on quantification is in the Supplementary Data).

PSD-95 clusters on dendrites are normalized to cluster density per 10 μm. Statistics were performed with R package ggpubr using unpaired *t*-test (*p ≤ 0.05; **p ≤ 0.01; ***p ≤ 0.001, ****p ≤ 0.0001). For 3D reconstruction images, Bitplane Imaris software 8.2 was used. iNs were transduced with pLVXTP-GFP and transferred to tissue culture-treated ibidi μ-slides coated with Geltrex^®^ Matrix Products. Confocal images were taken on Zeiss Cell Observer Spinning Disc Confocal Microscope.

### Calcium imaging

Following 21 days of conversion, iNs from CL05, CL12, and CL15 were isolated with fluorescence activated cell sorting (FACS) as previously described (Zhou-Yang et al., 2021) and replated on Geltrex-coated dishes. Media were changed to BrainPhys-based maturation media containing N2, B27, glial cell line-derived neurotrophic factor (GDNF), brain-derived neurotrophic factor (BDNF) (both 20 ng/mL, R&D), dibutyryl cyclic AMP (500 μg/mL, Sigma-Aldrich), doxycycline (2 μg/mL, Sigma-Aldrich), and laminin (1 μg/mL, Thermo Fisher Scientific). On day 25 of conversion, iNs were transduced with lentiviral particles for CAG::GcAMP6m. Calcium imaging was performed on day 35 of conversion on Zeiss Cell Observer Spinning Disc Confocal Microscope with 25 × objective, at 488 nm excitation and controlled temperature (37°C), and CO_2_ concentration (5%). Time-lapse images were captured using Hamamatsu camera at 2.5 frames per second. Cell lines were assessed with three fields per cell line. Analysis was performed as previously described (Mertens et al., 2021).

## Supplementary Material

Supplemental data

Supplemental data

Supplemental data

Supplemental data

Supplemental data

Supplemental data

Supplemental data

Supplemental data
