# Supplementary material for: Chemical Replacement of Noggin with Dorsomorphin Homolog 1 for Cost-Effective Direct Neuronal Conversion
Source: Cell Reprogram. 2022 Oct 7;24(5):304–13. doi: 10.1089/cell.2021.0200 (PMC9587801; doi:10.1089/cell.2021.0200)
Supplement: Supplemental data [file Suppl_TableS1.docx]

SupplTab.T1 Selected neuronal genes for PCA

| Gene |
| --- |
| CALB2 |
| CNIH2 |
| CNR1 |
| DRD2 |
| GABBR2 |
| GAD1 |
| GAD11 |
| GRIA2 |
| GRID2 |
| GRIK2 |
| GRIK5 |
| GRIN1 |
| MAP2 |
| NEFH |
| NEFL |
| NEUROD1 |
| PLCL2 |
| RBFOX3 |
| SLC17A7 |
| SLC6A1 |
| SYN1 |
| SYN2 |
| SYN3 |
| SYP |
| TUBB3 |
| UNC13A |
