# Supplementary material for: Chemical Replacement of Noggin with Dorsomorphin Homolog 1 for Cost-Effective Direct Neuronal Conversion
Source: Cell Reprogram. 2022 Oct 7;24(5):304–13. doi: 10.1089/cell.2021.0200 (PMC9587801; doi:10.1089/cell.2021.0200)
Supplement: Supplemental data [file Suppl_FigS1.docx]

Fig.S1

(A) PC loadings based on PCA of 26 selected neuronal genes. Highlighted: NEFL, TUBB3, GABBR2, GRIA2, RBFOX3.

(B) PCA analysis of differentially expressed genes (n=25,609) between iNs and FBs.

(C) PCA analysis of genes in Reactome pathway *Neuronal System* (R-HSA-112316) between iNs and FBs.

(D) PC loadings of top 50 rows in Reactome pathway *Neuronal System* (R-HSA-112316) between iNs and FBs.

(E-F) Enrichment plots of 3 enriched pathways in iN (E) and 3 enriched pathways in FBs (F).

(G) Gene expression of BMP signaling arm specific receptor genes ACVR1 (orange), ACVRL1 (dark blue), BMPR1A (turquoise) and BMPR1B (yellow) in FBs.
