# Supplementary material for: Chemical Replacement of Noggin with Dorsomorphin Homolog 1 for Cost-Effective Direct Neuronal Conversion
Source: Cell Reprogram. 2022 Oct 7;24(5):304–13. doi: 10.1089/cell.2021.0200 (PMC9587801; doi:10.1089/cell.2021.0200)
Supplement: Supplemental data [file Suppl_TableS2.docx]

SupplTab.T2

| ID | Description | setSize | enrichmentScore | NES | pvalue | p.adjust |
| --- | --- | --- | --- | --- | --- | --- |
| hsa04080 | Neuroactive ligand-receptor interaction | 315 | 0.49814533 | 1.79919455 | 0.00010135 | 0.00568717 |
| hsa05012 | Parkinson disease | 231 | 0.51107923 | 1.8177543 | 0.00010314 | 0.00568717 |
| hsa05415 | Diabetic cardiomyopathy | 173 | 0.4736397 | 1.6540571 | 0.00010549 | 0.00568717 |
| hsa03010 | Ribosome | 134 | 0.66063397 | 2.26445539 | 0.00010783 | 0.00568717 |
| hsa00190 | Oxidative phosphorylation | 104 | 0.64615165 | 2.15902714 | 0.00011106 | 0.00568717 |
| hsa04260 | Cardiac muscle contraction | 78 | 0.5589671 | 1.81303439 | 0.00011496 | 0.00568717 |
| hsa04721 | Synaptic vesicle cycle | 76 | 0.62055521 | 2.00319574 | 0.00011573 | 0.00568717 |
| hsa05016 | Huntington disease | 274 | 0.4295488 | 1.54238629 | 0.00030606 | 0.01179577 |
| hsa05020 | Prion disease | 239 | 0.44498693 | 1.58665309 | 0.00030861 | 0.01179577 |
| hsa04024 | cAMP signaling pathway | 207 | 0.4415105 | 1.5600382 | 0.00051937 | 0.01628958 |
| hsa04714 | Thermogenesis | 200 | 0.44190686 | 1.55862736 | 0.00052089 | 0.01628958 |
| hsa05320 | Autoimmune thyroid disease | 35 | 0.63226341 | 1.80532797 | 0.00064408 | 0.01798572 |
| hsa05208 | Chemical carcinogenesis - reactive oxygen species | 194 | 0.43851152 | 1.54231172 | 0.00083743 | 0.01798572 |
| hsa04512 | ECM-receptor interaction | 87 | -0.4239249 | -1.8009454 | 0.00084104 | 0.01798572 |
| hsa04350 | TGF-beta signaling pathway | 91 | -0.4100091 | -1.7500711 | 0.0008569 | 0.01798572 |
| hsa05146 | Amoebiasis | 93 | -0.38488 | -1.649047 | 0.00088496 | 0.01798572 |
| hsa05215 | Prostate cancer | 97 | -0.4085204 | -1.7659041 | 0.00093023 | 0.01798572 |
| hsa04966 | Collecting duct acid secretion | 26 | 0.66580005 | 1.79197892 | 0.00094111 | 0.01798572 |
| hsa05014 | Amyotrophic lateral sclerosis | 328 | 0.40214448 | 1.45492216 | 0.00101133 | 0.01831034 |
| hsa04940 | Type I diabetes mellitus | 33 | 0.62216408 | 1.75639184 | 0.00116974 | 0.01913769 |
| hsa04110 | Cell cycle | 125 | -0.399397 | -1.7892842 | 0.00126103 | 0.01913769 |
| hsa04068 | FoxO signaling pathway | 127 | -0.3577871 | -1.6105328 | 0.00127065 | 0.01913769 |
| hsa00982 | Drug metabolism - cytochrome P450 | 61 | -0.437377 | -1.7400756 | 0.00127959 | 0.01913769 |
| hsa04970 | Salivary secretion | 77 | 0.51619483 | 1.6698858 | 0.0013844 | 0.01913769 |
| hsa04630 | JAK-STAT signaling pathway | 139 | -0.4070907 | -1.8633877 | 0.00139082 | 0.01913769 |
| hsa00053 | Ascorbate and aldarate metabolism | 24 | -0.6060717 | -1.9588291 | 0.00189394 | 0.02500249 |
| hsa05171 | Coronavirus disease - COVID-19 | 217 | 0.4193651 | 1.4872051 | 0.0019624 | 0.02500249 |
| hsa04520 | Adherens junction | 66 | -0.4175775 | -1.6899756 | 0.00205058 | 0.02519285 |
| hsa00830 | Retinol metabolism | 54 | -0.4512136 | -1.7555651 | 0.00232558 | 0.02738817 |
| hsa04510 | Focal adhesion | 197 | -0.3599425 | -1.7226057 | 0.00242718 | 0.02738817 |
| hsa04964 | Proximal tubule bicarbonate reclamation | 22 | 0.67156229 | 1.74744163 | 0.00246812 | 0.02738817 |
| hsa05205 | Proteoglycans in cancer | 203 | -0.3105877 | -1.4913663 | 0.00258398 | 0.02777778 |
| hsa03040 | Spliceosome | 139 | 0.4428406 | 1.52064646 | 0.00290854 | 0.02967052 |
| hsa04810 | Regulation of actin cytoskeleton | 212 | -0.3820922 | -1.8543052 | 0.00293255 | 0.02967052 |
| hsa05206 | MicroRNAs in cancer | 221 | -0.3158775 | -1.5280464 | 0.00328947 | 0.03191018 |
| hsa04723 | Retrograde endocannabinoid signaling | 138 | 0.44066436 | 1.51294116 | 0.00333944 | 0.03191018 |
| hsa03320 | PPAR signaling pathway | 70 | 0.50583664 | 1.61580528 | 0.00373919 | 0.03476437 |
| hsa04072 | Phospholipase D signaling pathway | 139 | -0.307883 | -1.4092814 | 0.00417246 | 0.03777176 |
| hsa04625 | C-type lectin receptor signaling pathway | 98 | -0.3473863 | -1.5055627 | 0.00464684 | 0.04066194 |
| hsa04915 | Estrogen signaling pathway | 121 | -0.3311984 | -1.4810237 | 0.00472813 | 0.04066194 |
| hsa04925 | Aldosterone synthesis and secretion | 91 | 0.47218776 | 1.5554465 | 0.00543294 | 0.04558367 |
| hsa04911 | Insulin secretion | 81 | 0.47824406 | 1.55744867 | 0.00595511 | 0.04821092 |
| hsa04668 | TNF signaling pathway | 108 | -0.3296839 | -1.4556696 | 0.0061665 | 0.04821092 |
| hsa05150 | Staphylococcus aureus infection | 67 | -0.3753213 | -1.5221752 | 0.00617708 | 0.04821092 |
| hsa00040 | Pentose and glucuronate interconversions | 27 | -0.5208456 | -1.7404054 | 0.00630666 | 0.04821092 |
| hsa05161 | Hepatitis B | 154 | -0.3005285 | -1.3904994 | 0.00661157 | 0.04944305 |

ID = KEGG pathway ID; NES = normalized enrichment score; p.adjust = adjusted p-value (method: Benjamini-Hochberg);
