# Supplementary material for: Chemical Replacement of Noggin with Dorsomorphin Homolog 1 for Cost-Effective Direct Neuronal Conversion
Source: Cell Reprogram. 2022 Oct 7;24(5):304–13. doi: 10.1089/cell.2021.0200 (PMC9587801; doi:10.1089/cell.2021.0200)
Supplement: Supplemental data [file Suppl_FigS2.docx]

Fig.S2

(A) Concentration curve of +DMH1 media with different concentrations of DMH1 (red). Green line indicates percentage of βIII-tubulin cells over DAPI in +Noggin media. Saturation is reached at 5 µM DMH1(dotted blue line).

(B) Brightfield images of CL01 before (D-1) and after (D0) pool split, and during (D7, D14, D21) conversion in three different neuronal conversion media (tSMADi, +Noggin, +DMH1). Scale bars: 50 µm.
