# Supplementary material for: Chemical Replacement of Noggin with Dorsomorphin Homolog 1 for Cost-Effective Direct Neuronal Conversion
Source: Cell Reprogram. 2022 Oct 7;24(5):304–13. doi: 10.1089/cell.2021.0200 (PMC9587801; doi:10.1089/cell.2021.0200)
Supplement: Supplemental data [file Suppl_FigS3.docx]

Fig.S3

(A) Immunofluorescent images of CL02 on 7, 14 and 21 days of conversion. βIII-tubulin (red), DAPI (blue).

(B) Immunofluorescent images of CL02 on 7, 14 and 21 days of conversion. NeuN (green), DAPI (blue).

Scale bars: 50 µm.
