# Supplementary material for: Chemical Replacement of Noggin with Dorsomorphin Homolog 1 for Cost-Effective Direct Neuronal Conversion
Source: Cell Reprogram. 2022 Oct 7;24(5):304–13. doi: 10.1089/cell.2021.0200 (PMC9587801; doi:10.1089/cell.2021.0200)
Supplement: Supplemental data [file Suppl_TableS4.docx]

SupplTab.T4

| Name | Age [years] | Sex |
| --- | --- | --- |
| CL01 | 68 | Female |
| CL02 | 77 | Female |
| CL03 | 55 | Female |
| CL04 | 81 | Female |
| CL05 | 79 | Female |
| CL06 | 55 | Male |
| CL07 | 79 | Female |
| CL08 | 67 | Female |
| CL09 | 74 | Male |
| CL10 | 64 | Female |
| CL11 | 55 | Female |
| CL12 | 66 | Male |
| CL13 | 66 | Male |
| CL14 | 59 | Male |
| CL15 | 56 | Male |
