# Supplementary material for: Chemical Replacement of Noggin with Dorsomorphin Homolog 1 for Cost-Effective Direct Neuronal Conversion
Source: Cell Reprogram. 2022 Oct 7;24(5):304–13. doi: 10.1089/cell.2021.0200 (PMC9587801; doi:10.1089/cell.2021.0200)
Supplement: Supplemental data [file Suppl_FigS4.docx]

Fig.S4

(A) Percentage of whole of each component of +Noggin medium.
