# Supplementary material for: Chemical Replacement of Noggin with Dorsomorphin Homolog 1 for Cost-Effective Direct Neuronal Conversion
Source: Cell Reprogram. 2022 Oct 7;24(5):304–13. doi: 10.1089/cell.2021.0200 (PMC9587801; doi:10.1089/cell.2021.0200)
Supplement: Supplemental data [file Suppl_Data.docx]

Supplementary Data

Method: Quantification of IF data

All fluorescence images were acquired with an inverse microscope Leica DMi8, and exact settings were used for acquiring each image, in terms of exposure and magnification. For each cell line and condition, three independent replicates were carried out. For image analysis, a FIJI software was used, three images per cell line per condition were taken, and at least 30 cells per image was included for analysis.

Induced neurons, in comparison to other neuronal cultures e.g. iPSC-derived neurons, are not prompt for cluster formation in culture. For this reason, each independent neuron is easily identified and can be counted manually with high accuracy. Every image was counted manually by the authors without using an automated software for cell counting, and each count is normalized to DAPI to exclude miss-differentiated or intermediate cells that do not express TUJ1. The mean and the standard deviation were calculated using GraphPad Prism 8.4.3 software. A t-test was performed to determine whether a significant difference exists between groups (* p <= 0,05; ** p <= 0,01; *** p <= 0,001).
